# Parameter-Dependent Electroporation and Cellular Responses Induced by Microsecond Pulsed Electric Fields: Different Sensitivity of Two Pancreatic Ductal Adenocarcinoma Cell Lines

**DOI:** 10.3390/bioengineering13091056

**Published:** 2026-09-11

**Authors:** Mariateresa Allocca, Stefania Romeo, Maria Rosaria Scarfì, Olga Zeni, Anna Sannino, Giuseppina Palmiero, Sanober Kafeel, Angela Ragone, Silvio Naviglio, Luigi Sapio

**Affiliations:** 1 Institute for Electromagnetic Sensing of the Environment (IREA), CNR, Via Diocleziano 320, 80124 Napoli, Italy; allocca.m@irea.cnr.it (M.A.); scarfi.mr@irea.cnr.it (M.R.S.); zeni.o@irea.cnr.it (O.Z.); sannino.a@irea.cnr.it (A.S.); 2 Department of Precision Medicine, University of Campania “Luigi Vanvitelli”, Via L. De Crecchio 7, 80138 Napoli, Italy; giuseppina.palmiero@unicampania.it (G.P.); sanober.kafeel@unicampania.it (S.K.); angela.ragone@unicampania.it (A.R.); silvio.naviglio@unicampania.it (S.N.); luigi.sapio@unicampania.it (L.S.); 3 Department of Mechanistic Cell Biology, Max Plank Institute of Molecular Physiology, 44227 Dortmund, Germany

**Keywords:** reversible electroporation, irreversible electroporation, pancreatic ductal adenocarcinoma cells, pulsed electric fields, cellular stress response

## Abstract

Pancreatic ductal adenocarcinoma (PDAC) is a highly aggressive, treatment-resistant tumor requiring novel therapeutic approaches. Electroporation (EP)-based strategies are promising tools for local tumor control and enhanced drug delivery. However, cellular responses and protocols optimization remain poorly investigated in PDAC. Here, we investigated the effects of variable microsecond pulsed electric fields (PEFs) on PANC-1 and MIA PaCa-2 PDAC cells. Immediate responses were assessed via membrane permeabilization and cell death. Delayed effects were evaluated through viability, growth, cell cycle and expression of stress markers. Immediate cell death occurred above specific thresholds in PANC-1 and MIA PaCa-2 cells (16 pulses at 1 kV/cm and 30 pulses at 2.5 kV/cm, respectively), suggesting differential sensitivity to PEFs. Delayed analyses confirmed differences in both viability (−19.3% vs. −46.4% with 4 pulses, 1 kV/cm, 48 h) and growth (−30.3% vs. −57.6% with 4 pulses, 1 kV/cm, 48 h). An opposite modulation of the S-phase population was also observed at 48 h (+7.4% with 20 pulses at 1 kV/cm and −9.2% with 8 pulses at 1 kV/cm for PANC-1 and MIA PaCa-2, respectively). Both cell lines showed similar regulations of apoptosis-associated and autophagy-related markers. Overall, PEFs elicited parameter- and cell-specific responses, supporting fine-tuned EP protocols for pancreatic cancer therapy.

## 1. Introduction

Electroporation (EP) is a biophysical phenomenon characterized by an increase in cell membrane permeability induced by the application of short, high-intensity pulsed electric fields (PEFs), which cause the transmembrane voltage to exceed a critical threshold. Depending on the electric field strength reached during EP, cells may either restore membrane integrity and survive (Reversible EP, REP), or undergo cell death as a consequence of extensive, non-repairable damage (Irreversible EP, IRE). REP is used in biotechnology and medicine to facilitate the intracellular delivery of otherwise impermeant molecules, such as nucleic acids, or to enhance the uptake of therapeutic agents, as exploited in electrochemotherapy (ECT) [1]. Conversely, IRE is a focal, non-thermal ablative technique employed in clinical practice for treating cardiovascular and malignant diseases, particularly in cases unsuitable for surgery or thermal ablation, such as pancreatic and liver cancers [2]. Most clinical REP and IRE applications rely on pulses in the microsecond to millisecond time range, while the biological outcome is mainly determined by differences in electric field strength, pulse number, duration and repetition rate [3].

Despite the recognized advantages of REP and IRE, the complex relationships between PEF parameters, membrane permeabilization, and subsequent cellular responses are still not completely understood. Previous studies have demonstrated that EP-induced cell death is a dynamic process that depends not only on pulse characteristics but also on the intrinsic properties of the target cells and on the post-treatment observation time [1,3]. A better understanding of how different cell models respond to specific PEF conditions is therefore essential for predicting and controlling EP-based responses. In particular, the identification of parameter-dependent thresholds associated with membrane permeabilization, recovery, and loss of viability may provide useful information for optimizing EP protocols in different biological contexts.

Pancreatic ductal adenocarcinoma (PDAC) is a highly aggressive disease, accounting for almost 90% of all diagnosed pancreatic tumors, and remains one of the leading causes of cancer-related mortality worldwide. Most patients with pancreatic cancer present nonspecific symptoms at an advanced stage of the disease, when curative surgery is no longer feasible [4]. The intrinsic aggressive features and high resistance to conventional chemotherapy, as well as the high recurrence rate, make PDAC particularly hard to treat [5]. Surgical resection remains the only potentially curative treatment; however, only a limited proportion of patients are eligible at diagnosis because of local vascular involvement and the anatomical complexity of the pancreatic region. Chemotherapy and radiotherapy are also applied, but advances in targeted therapy and immunotherapy are opening new possibilities [6]. These limitations highlight the need for the development and characterization of innovative approaches for the management of PDAC [7,8,9].

Both ECT and IRE have been proposed as innovative therapeutic modalities for local tumor control in PDAC, potentially in combination with other pharmacological or physical treatments [10,11]. REP is being investigated in locally advanced pancreatic cancer to improve local tumor control, whereas IRE can be used in patients with nonresectable pancreatic tumors who are not candidates for surgery [9]. Despite the growing interest in EP-based interventions for pancreatic cancer, the relationships among PEF parameters, membrane permeabilization, and long-term survival remain incompletely characterized in PDAC models.

To address this knowledge gap, the present study was designed with a twofold purpose: (i) to investigate how different PEF parameters influence cellular responses in PDAC cells; (ii) to explore how different PDAC cells react under identical PEF stimulation. To achieve these aims, two widely used human PDAC cell lines (PANC-1 and MIA PaCa-2) were exposed to a broad range of PEF conditions, and their immediate and delayed responses were subsequently analyzed. Initial screening experiments were performed to evaluate membrane permeabilization and cell viability after PEFs exposure using both flow cytometric and colorimetric assays. Based on the results from the screening phase, selected PEF conditions representative of mild, intermediate, and intense EP were further investigated for the delayed effects on cell growth and cell-cycle progression by colorimetric and flow cytometric assays. In addition, molecular analyses by Western blotting were conducted to gain insight into the cellular mechanisms underlying EP-induced effects.

## 2. Materials and Methods

### 2.1. Reagents and Antibodies

Dulbecco’s Minimum Essential Medium (DMEM), Fetal bovine Serum (FBS), Penicillin-Streptomycin, L-glutamine, Trypsin-EDTA and Phosphate-Buffered Saline (PBS) were from EuroClone (Pero, Italy). Calcein acetoxymethyl ester (Calcein-AM, CAM), Propidium Iodide (PI), (3-(4,5-dimethylthiazol-2-yl)-2,5 diphenyltetrazolium bromide) (MTT), RNaseA, HEPES, sucrose, magnesium chloride (MgCl_2_), calcium chloride (CaCl_2_) and RIPA buffer solution were from Sigma (St. Louis, MO, USA). Trypan blue was from Gibco (Waltham, MA, USA). Protease and phosphatase inhibitor cocktail were from Cell Signaling (Danvers, MA, USA). Ponceau S, Bradford solution, Bovine serum albumin (BSA), Sodium dodecyl sulfate, Sodium Chloride, Tris Base and non-fat dry milk were from PanReac AppliChem (Chicago, IL, USA). Laemmli buffer and Potassium Chloride were from Fisher Scientific (Waltham, MA, USA). Amersham Protran 0.45 nitrocellulose membrane were from Cytiva (Marlborough, MA, USA). Excellent chemiluminescent substrate kit was from Elabsciences (Houston, TX, USA). LDH Reagent Kit was from Abbott, Lake County, IL, USA). Ethanol, Isopropanol, and HCl were from Carlo Erba (Cornaredo, Italy). Tween 20 was from SERVA (Heidelberg, Germany).

The following primary antibodies were employed for Western blotting detection: Cyclin A (H-432) (sc-751) and SQSTM1/p62 (sc-28359) were purchased from Santa Cruz Biotechnology (Dallas, TX, USA); Cleaved Caspase-3 (Asp175) (5A1E) (#9664), Poly (ADP-ribose) polymerase (PARP) (#9542), Vinculin (#13901), β-Actin (8H10D10) (#3700), α-Tubulin (DM1A) (#3873), p21 Waf1/Cip1 (12D1) (#2947), LC3B (#2775), Cyclin D1 (#55506), p27 Kip1 (#3686), goat antirabbit IgG HRP-linked (#7074), and goat anti-mouse IgG HRP-linked (#7076) were obtained from Cell Signaling Technology (Danvers, MA, USA).

### 2.2. Cell Models and Handling

PANC-1 (CRL-1469) and MIA PaCa-2 (CRL-1420) cell lines (ATCC, Manassas, VA, USA) were cultured in DMEM supplemented with 10% heat-inactivated FBS, 1% Penicillin-Streptomycin, and 1% L-Glutamine. Cells were maintained at 37 °C in a humidified atmosphere containing 95% air and 5% CO_2_ in a commercial incubator (Forma Scientific, model 311, Freehold, NJ, USA) and were routinely checked for mycoplasma contamination. Cultures were supplied with fresh culture medium every 48 h and kept exponentially growing by splitting them once a week by trypsin treatment (1× trypsin-EDTA solution for 5 min). For consistency and reproducibility across experiments, a master bank of cells was established, and the same batch of reagents was used throughout the study. All experiments were performed using cells between passages 12 and 20.

### 2.3. Experimental Protocol and Pulsing Conditions

The ELECTROcell-B15 high voltage pulse generator (Leroy Biotech, Saint-Laurent-Blangy, France) was used to deliver microsecond electric pulses to cell samples hosted in conventional 4 mm EP cuvettes (Sigma Aldrich, St. Louis, MO, USA).

The pulse generator and EP cuvettes were characterized by voltage and current measurements performed using different pulsing buffers: namely, DMEM and a low-conductivity (201 μS/cm) pulsing buffer (10 mM HEPES, 250 mM sucrose, 1.5 mM MgCl_2_, 0.6 mM CaCl_2_) [12], as well as different sample volumes. The measurements were performed to verify voltage stability and compliance with the generator current threshold. For each independent experiment, cells were harvested from the same parental flask. For each experimental condition, 1 × 10^6^ cells were suspended in 400 μL of low-conductivity pulsing buffer and exposed to the electric pulses. This buffer composition allowed stable pulse delivery over the entire voltage range investigated while maintaining the current within the compliance limits of the pulse generator.

High voltage, 100-μs-long electric pulses, and 1-Hz repetition rate were applied with a variable number of pulses (1–50) and voltages ranging from 400 V to 1000 V (corresponding to voltage-to-distance ratios from 1.0 to 2.5 kV/cm in a 4-mm EP cuvette). When more than 1 × 10^6^ cells were required for a specific analysis, cells exposed under identical pulsing conditions in independent cuvettes were pooled and seeded according to the experimental procedures. For each cell type/assay, an exposed sample was paired with a sham-exposed control (0 pulses) consisting of cells suspended in pulsing buffer and loaded into the EP cuvette without any application of electric pulses. A summary of the specific pulsing conditions adopted for each assay in the PDAC cell lines is presented in Table 1. Flow-cytometry (CAM/PI) and colorimetric (MTT) assays were used to investigate cellular responses across a broad range of electrical doses, encompassing conditions expected to induce responses ranging from limited membrane permeabilization to extensive EP-associated cell damage. PEF conditions representative of mild, intermediate, and intense EP were subsequently identified and applied to assess their effects on cell growth, cell-cycle progression, and cellular stress mechanisms associated with EP-induced damage and cell death.

### 2.4. Analysis of Immediate Electroporation Outcomes

To assess the immediate effects of EP following pulse application, a flow-cytometric assay was performed using CAM and PI staining. CAM is an esterified anionic fluorochrome that enters viable cells freely and becomes trapped in the cytoplasm by an intact cell membrane following the removal of the AM group. PI is a membrane impermeant dye, generally excluded from viable cells, which passes through permeabilized membranes and fluoresces upon binding to nucleic acids. PI uptake provides information on membrane permeabilization and, when combined with CAM staining, allows discrimination between viable permeabilized cells and non-viable cells [12]. For experiments, cells were exposed to PEFs in the presence of PI (40 μM) and then incubated for 5 min at room temperature (RT) to allow dye uptake by permeabilized cells. Subsequently, samples were washed in PBS and incubated at RT in PBS containing 0.2 μM of CAM using a 10- or 30-min incubation time for PANC-1 and MIA PaCa-2 cells, respectively. A BD FacsCalibur flow cytometer (Becton Dickinson, San José, CA, USA; 488 nm excitation wavelength) was used to process the samples. For each sample, 20,000 events were acquired using CELL QUEST software v3.3 (Becton Dickinson, San José, CA, USA), and the raw fluorescence data were then quantitatively analyzed with the FlowJo analysis program (TreeStar, Woodburn, OR, USA). The percentage of dead, permeabilized and viable cells was calculated as:

$$\%Dead_{cells}=100\times \frac{Q_{1}}{Q_{1}+Q_{2}+Q_{3}}$$

$${\%Permeabilized}_{cells}=100\times \frac{Q_{2}}{Q_{1}+Q_{2}+Q_{3}}$$

$${\%Viable}_{cells}=100\times \frac{Q_{3}}{Q_{1}+Q_{2}+Q_{3}}$$

where Q1, Q2 and Q3 are, respectively, the CAM^−^/PI^+^ (dead cells), CAM^+^/PI^+^ (viable and permeabilized cells), and CAM^+^/PI^−^ (viable and non-permeabilized cells) quadrants of the flow-cytometry dot plot. Unstained debris and non-cellular events (falling within the Q4: CAM^−^/PI^−^ quadrant) were excluded from the analysis.

### 2.5. Analysis of Delayed Cytotoxic Effects

#### 2.5.1. MTT Assay

To assess the mitochondrial metabolic activity, the MTT assay (3-(4,5-dimethylthiazol-2-yl)-2,5 diphenyltetrazolium bromide) was employed. Briefly, PANC-1 and MIA PaCa-2 cells were exposed or sham-exposed to different numbers of pulses, and 1.5 × 10^4^ cells/well were subsequently seeded in 96-well plates. At 24 and 48 h after treatment, 10 μL of MTT solution (1 mg/mL) was added to each well, allowing the formation of MTT formazan crystals during a 3-h incubation at 37 °C. After medium removal, the crystals were dissolved in 100 μL of Isopropanol-HCl 0.04 N by shaking the plate for 20 min at RT. An Infinite 200 PRO Microplate Reader (Tecan Life Sciences, Männedorf, Switzerland) was used to measure the optical density of each sample at 570 nm.

#### 2.5.2. Trypan Blue Exclusion Method

The Trypan blue dye exclusion method (TBEM) was applied to discriminate living and dead cells based on the principle that the dye enters cells with compromised plasma membrane integrity. After exposure to different pulsing conditions, PANC-1 and MIA PaCa-2 cells were seeded in a 6-well plate at densities of 3 × 10^5^ and 2 × 10^5^ cells/well, respectively. At 24 and 48 h post-seeding, cells were detached using 1× Trypsin-EDTA, and 10 μL of the cell-containing medium was mixed with an identical Trypan blue volume. The relative numbers of live (unstained) and dead (blue-stained) cells were determined by automated cell counting (Corning^®^ Cell Counter, Corning, NY, USA). The fraction of dead cells was also expressed as percentage of the total number of cells.

#### 2.5.3. Lactate Dehydrogenase Assay

Lactate dehydrogenase (LDH) enzymatic activity was evaluated in exhaust media using LDH Reagent Kit (7D69, Abbott, Lake County, IL, USA). As a stable cytosolic enzyme involved in anaerobic glycolysis, LDH catalyzes the conversion of lactate to pyruvate with the reduction of NAD+ to NADH and vice versa. Therefore, the release of LDH into the surrounding medium is usually associated with the loss of membrane integrity and can thus be used as a direct marker of cell death. In detail, culture medium was collected and centrifuged (1200 rpm, 5 min) at 24 and 48 h post-pulse. Then, the resulting supernatant was analyzed for LDH enzymatic activity through the spectrophotometric measurement of NADH at 340 nm by Abbott Architect ci8200 analyzer (Abbott, Lake County, IL, USA).

### 2.6. Analysis of Mechanistic Responses

#### 2.6.1. Cell Cycle Analysis

PI staining was used to evaluate the DNA content of permeabilized cells, and cell cycle profiles were obtained by flow cytometry. Following PEF exposure, PANC-1 and MIA PaCa-2 were seeded in a 6-well plate at densities of 3 × 10^5^ and 2 × 10^5^ per well, respectively. After 24- and 48-h cells were collected by trypsinization and centrifugation (1200 rpm, 5 min), the resulting pellets were washed in PBS and resuspended in a solution of 70% ice-cold absolute ethanol/PBS. Permeabilized cells were stored at −20 °C until analysis. After centrifugation (1200 rpm, 5 min), samples were dissolved in PBS solution containing 15 µg/mL of PI plus 20 µg/mL of RNaseA. The cell cycle profile was recorded by FACSCalibur, acquiring at least 20,000 events for each sample. Data analysis was carried out with ModFIT v.3.0 (Verity Software House, Topsham, ME, USA).

#### 2.6.2. Protein Extraction and Quantification

PEF-exposed/sham-exposed PANC-1 and MIA PaCa-2 cells were cultured into 100-mm Petri dishes at densities of 1.5 × 10^6^ and 1 × 10^6^ per dish, respectively. Cells were collected by scraping and centrifugation at 24 and 48 h post-pulse (1200 rpm, 5 min). The resulting pellets were stored at −80 °C until protein extraction and quantification. Once thawed, the pellets were lysed using a solution of RIPA Buffer and Protease and Phosphatase Inhibitor Cocktail. Samples were kept on ice for 30 min and then spun down at 12,000 rpm for 20 min at 4 °C. The supernatant was harvested, and the protein content of each sample was quantified using the Bradford method. Absorbance was recorded at 595 nm using UV/VIS Spectrophotometer V-550 (JASCO, Mary’s Court Easton, MD, USA) for both samples and BSA, the latter used as a standard for the calibration curve.

#### 2.6.3. Western Blotting Analysis

Samples for Western blotting analysis were prepared by mixing Laemmli buffer with protein extract at a ratio of 1:6. Protein denaturation was achieved by heating the samples at 95 °C for 6 min. Typically, an equal protein concentration for each sample (ranging from 20 to 30 μg depending on the protein target) was loaded and resolved by sodium dodecyl sulfate-polyacrylamide gel electrophoresis at 135 V. Proteins were then transferred to a nitrocellulose membrane by a Mini Trans-Blot (Bio-Rad Laboratories; Hercules, CA, USA) at 100 V for 90 min. To verify transfer efficiency, Ponceau S was used to stain proteins on the membranes. Following three washes with tris-buffered saline containing 0.05% Tween^®^ 20 (TBS-T), the membrane was blocked with TBS-T containing non-fat dry milk (5% *w*/*v*) for 1 h under gentle shaking. The membrane was then probed overnight at 4 °C with the primary antibodies of interest. The day after, membranes were washed with TBS-T and incubated for 1 h with anti-species HRP-conjugated secondary antibody at RT. The immunoreactive bands were visualized using an Excellent Chemiluminescent Substrate Kit and captured using a ChemiDoc XRS+ System (Bio-Rad Laboratories, Hercules, CA, USA). ImageJ software (Version 1.52a) was used for the densitometric analysis of the detected bands.

### 2.7. Statistical Analysis

CAM/PI assay data were evaluated by one-way analysis of variance (ANOVA) followed by Tukey’s test, performed in MATLAB R2019b (MathWorks), comparing all pulsing conditions between each other. A *p*-value < 0.05 was considered indicative of statistical significance.

For all other biological assays, Welch’s *t*-test was applied to address the significance of each pulsing condition compared with the sham (0 pulses), using GraphPad Prism Software (Version 8.0.2).

In all cases, results from at least three independent experiments were plotted on graphs as mean ± standard deviation, with the exact number of independent experiments provided in the figure captions. A *p*-value < 0.05 was considered significant and graphically indicated as: * *p* < 0.05, ** *p* < 0.01, *** *p* < 0.001, **** *p* < 0.0001.

## 3. Results

### 3.1. PANC-1 and MIA PaCa-2 Cells Exhibit Distinct Immediate Responses to PEF Exposure

**Figure 1:**
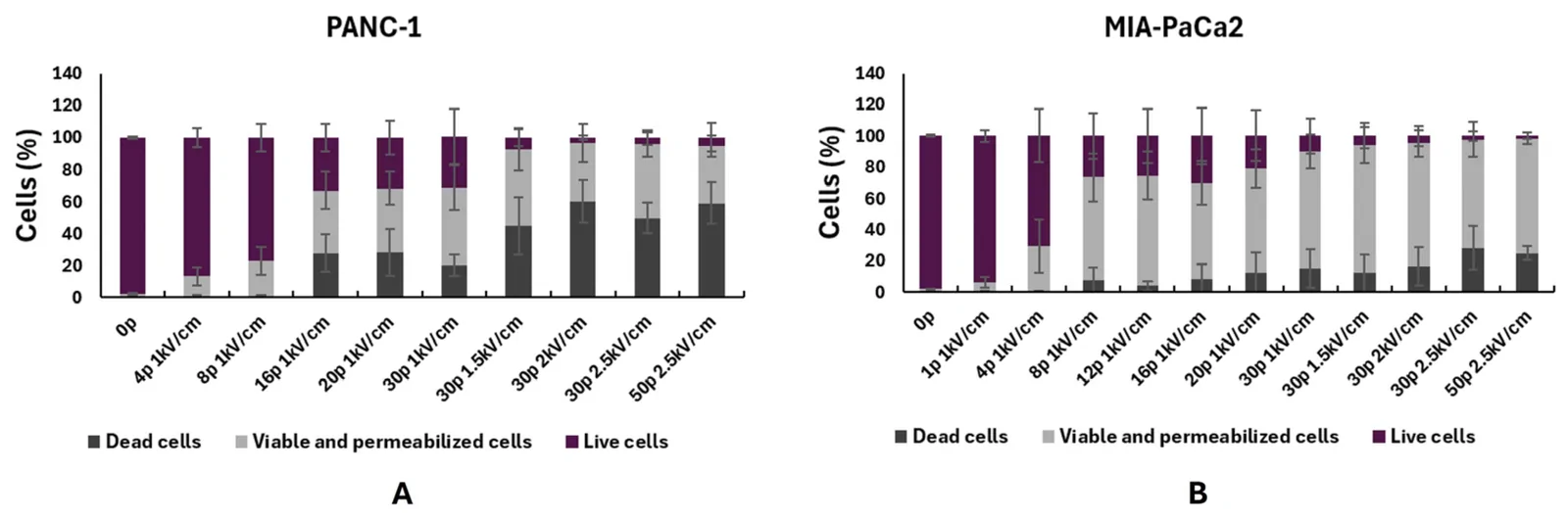
Immediate effects of pulsed electric fields. Stacked bar charts presenting, for PANC-1 (**A**) and MIA PaCa-2 (**B**) cells, the relative contribution of dead (Q1), viable and permeabilized (Q2), and live (Q3) cells for all the tested pulsing conditions, obtained by flow cytometry following CAM/PI staining. Data are reported as mean ± SD of at least four independent experiments.

The stacked bar charts in Figure 1 show, for each PEF condition, the relative contribution of the three cell populations identified by CAM/PI staining (Q1, Q2, and Q3) to the total number of acquired cells (excluding debris) in PANC-1 (Figure 1A) and MIA PaCa-2 (Figure 1B) cells.

In PANC-1 cells, the percentage of dead cells (Q1) became significantly higher than that of sham-exposed controls (0 p) when at least 16 pulses at 1 kV/cm were applied (*p* = 0.007, 16 p–1 kV/cm vs. sham). No additional increase in cell death was observed with 20 or 30 pulses at the same field strength. A further significant increase was detected following 30 pulses at 1.5 kV/cm (*p* = 0.007, 16 p–1 kV/cm vs. 30 p–1.5 kV/cm), after which the effect reached a plateau despite further increases in pulse number and/or voltage amplitude (Figure 1A and Figure S1A). The proportion of viable and permeabilized cells (Q2) increased significantly compared with the sham control starting from 8 pulses at 1 kV/cm (*p* = 0.02, 8 p–1 kV/cm vs. sham). Among the tested conditions, only 30 p–1, 2 and 2.5 kV/cm induced a further significant increase in permeabilization (*p* = 0.0002, 8 p–1 kV/cm vs. 30 p–1 kV/cm) (Figure 1A and Figure S1B). Conversely, the percentage of viable cells (Q3) progressively decreased with increasing pulse number, although not linearly, displaying a complementary trend to that observed for dead cells (Figure 1A and Figure S1C).

In MIA PaCa-2, the percentage of dead cells (Q1) became significantly higher than that of the sham after application of 30 p–1 kV/cm (*p* = 0.03, 30 p–1 kV/cm vs. sham) or subsequent conditions, except for 30 p–1.5 kV/cm (Figure 1B and Figure S1D). In contrast, the percentage of viable and permeabilized cells increased significantly at 4 p–1 kV/cm (*p* = 0.02, 4 p–1 kV/cm vs. sham) and reached a plateau at 8 p–1 kV/cm (*p* = 0.00001, 8 p–1 kV/cm vs. 4 p–1 kV/cm), with a small but significant further increase at 30 p–1.5 kV/cm (*p* = 0.003, 30 p–1.5 kV/cm vs. 8 p–1 kV/cm) (Figure 1B and Figure S1E). In this cell line, the trend observed for viable cells (Q3) was complementary to that of viable and permeabilized cells (Figure 1B and Figure S1F).

Overall, MIA PaCa-2 cells exhibited membrane permeabilization at lower pulse numbers than PANC-1 cells, whereas the onset of cell death required more intense exposure conditions in both cell lines.

The results of the one-way ANOVA test followed by Tukey post-hoc test are reported in Figure S1, panels A–C and D–F, for PANC-1 and MIA PaCa-2 cells, respectively.

### 3.2. PEFs Reduce Viability of PDAC Cells in a Pulse Number-Dependent Manner

To evaluate the delayed effects of different PEF conditions in PDAC cells, PANC-1 and MIA PaCa-2 were exposed to an increasing number of pulses (from 0 to 30) at 1 kV/cm and subsequently analyzed to assess mitochondrial activity as an indicator of post-treatment viability at 24 and 48 h.

**Figure 2:**
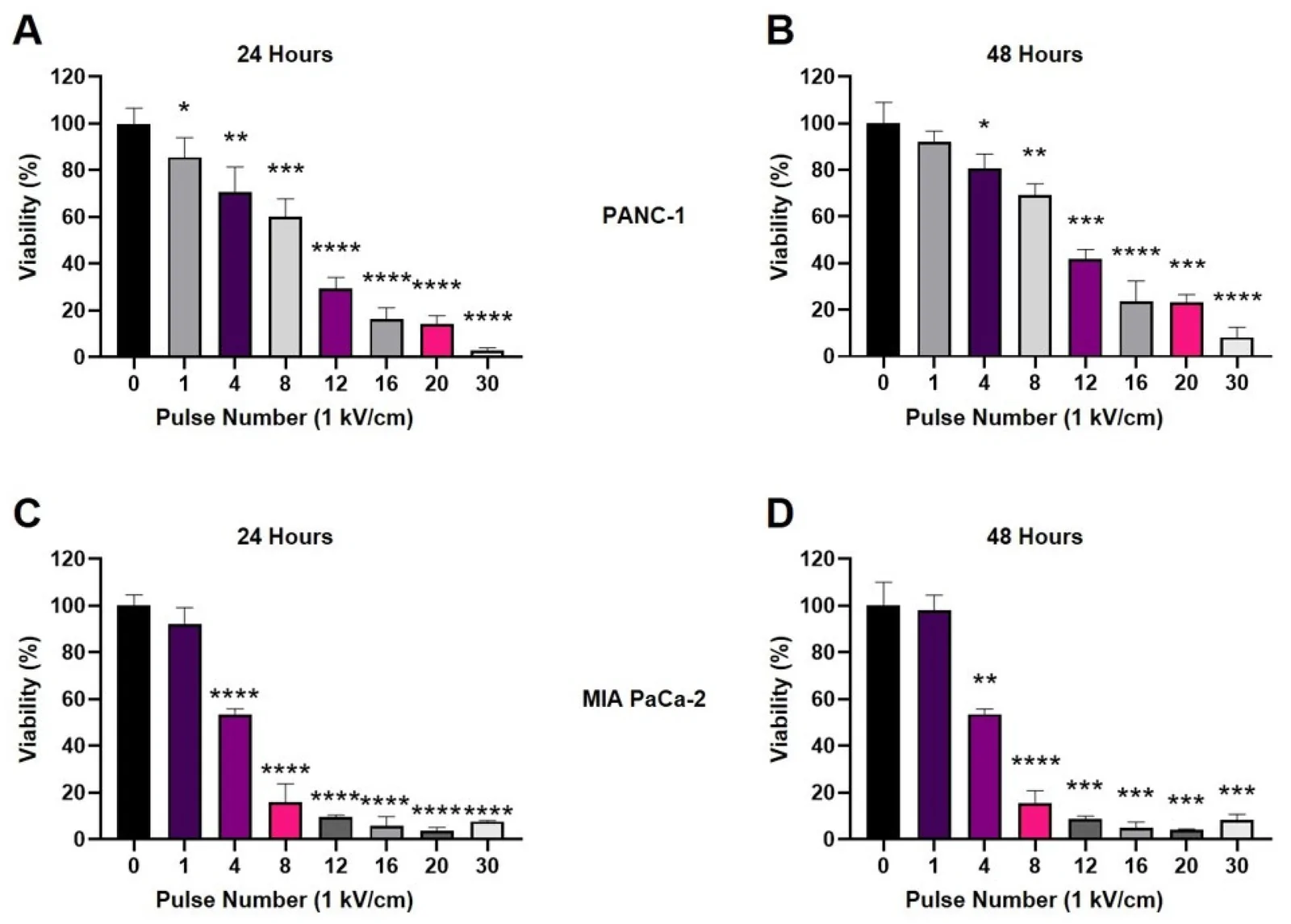
Evaluation of the pulse-mediated effects on PDAC cell viability by MTT assay. PANC-1 (**A**,**B**) and MIA PaCa-2 (**C**,**D**) cells were exposed to an increasing number of pulses (from 0 to 30), and viability was measured after 24 and 48 h. Results are reported as mean value ± SD of at least three independent experiments and expressed as % of sham control (0 pulses). * *p* < 0.05, ** *p* < 0.01, *** *p* < 0.001, **** *p* < 0.0001 by Welch’s *t*-test vs. sham (0 pulses).

The gradual increase in pulse number elicited a significant reduction in PANC-1 viability (Figure 2A,B). A statistically significant reduction was recorded for all the experimental conditions tested compared to the sham control in these cells, with viability falling below 50% after 12 pulses at 24 h. The outcome was even stronger when 30 pulses were applied, with no more than 5% of viable cells detected (Figure 2A). A comparable trend was observed at 48 h with only minor differences in magnitude (Figure 2B).

MIA PaCa-2 cells displayed an even greater susceptibility to PEFs compared to PANC-1. A significant reduction in metabolic activity was already observed after 4 pulses at 24 h, whereas exposure to 8 pulses reduced viability below 20% (Figure 2C). Both trend and magnitude were maintained at 48 h (Figure 2D).

Additional experiments performed at higher voltage-to-distance ratios (1.5 and 2.5 kV/cm) combined with 30 pulses showed comparable reductions in metabolic activity to those observed at 1 kV/cm. Detailed statistical comparisons are reported in Figure 2. Overall, these results indicate that PEFs affect the viability of PDAC cells in a pulse number-dependent manner, with MIA-PaCa-2 cells showing greater sensitivity than PANC-1 cells.

### 3.3. PEFs Impair PDAC Cell Growth by Reducing Live and Increasing Dead Cells

The effects of PEF exposure at 1 kV/cm were further evaluated by quantifying the number of viable and dead cells at 24 and 48 h post-treatment using TBEM. Considering the different susceptibility between PANC-1 and MIA PaCa-2, three pulsing conditions were selected from the broader range tested with MTT to distinguish mild (likely reversible) from extensive (likely irreversible) effects, depending on the cell line. Specifically, PANC-1 cells were exposed to 4, 12, and 20 pulses, whereas MIA PaCa-2 cells were exposed to 1, 4, and 8 pulses.

**Figure 3:**
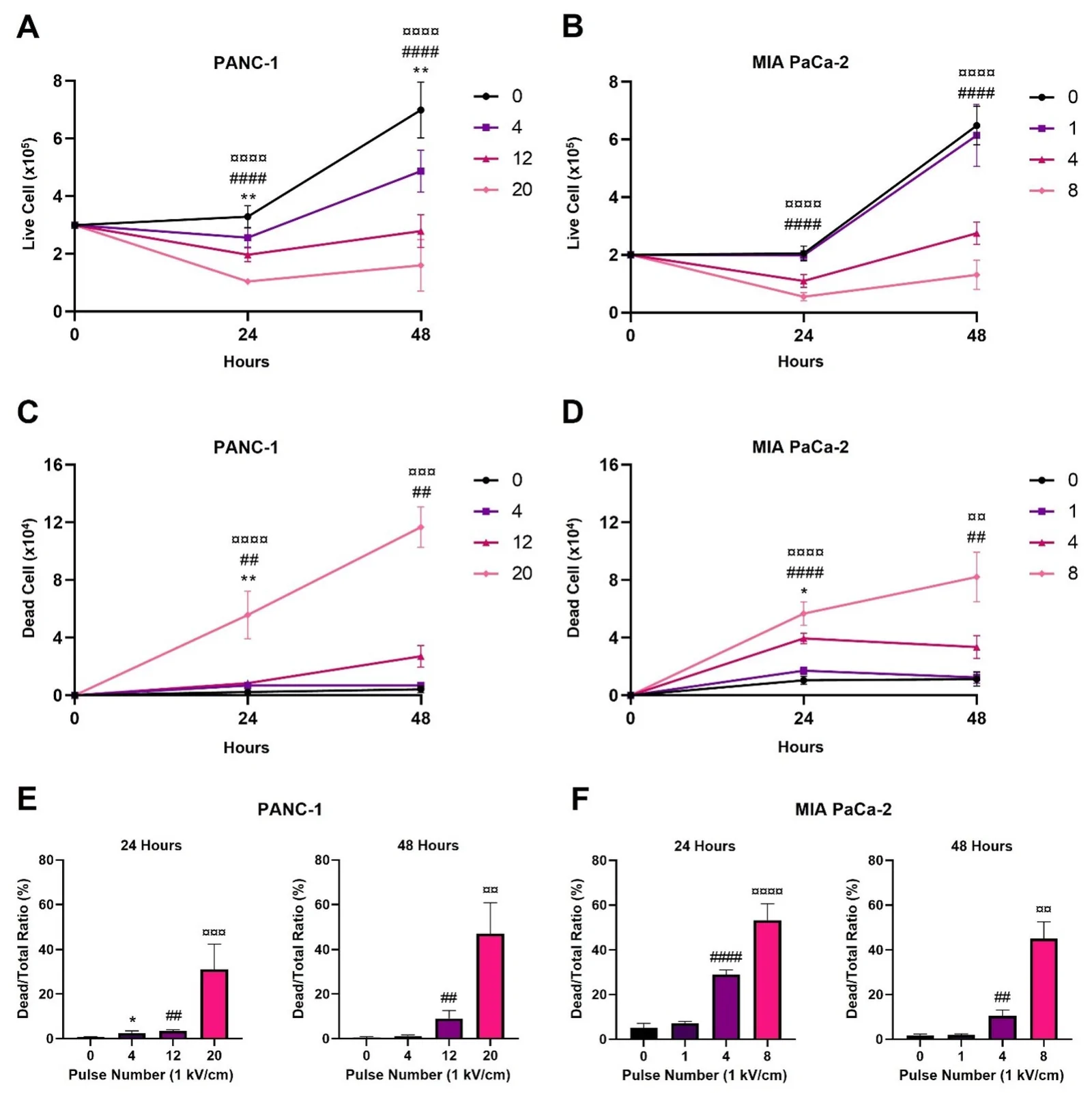
Investigation of the pulse-mediated effects on PDAC cell growth. PANC-1 cells were stimulated with 4, 12, and 20 pulses; thereafter, the number of living (**A**) and dead (**C**) cells was estimated at both 24 and 48 h. Similarly, MIA PaCa-2 cells were exposed to 1, 4 and 8 pulses before the numbers of living (**B**) and dead (**D**) cells were determined at 24 and 48 h (the legends of panels A to D report the number of applied pulses). The percentage of dead-to-total cell ratio was calculated in PANC-1 (**E**) and in MIA PaCa-2 (**F**) cells. * *p* < 0.05, ** ## ¤¤ *p* < 0.01, ¤¤¤ *p* < 0.001; #### ¤¤¤¤ *p* < 0.0001 by Welch’s *t*-test vs. sham (0 pulses).

Figure 3A displays a sharp decrease in the number of living cells 24 h after PEFs exposure compared to the sham control (0 pulses) in PANC-1 cells. This effect was even stronger 24 h later, where reductions of 30%, 60% and 77% were observed with 4, 12 and 20 pulses, respectively (Figure 3A). A similar drop was also recorded in MIA PaCa-2 with 4 (−57% vs. sham) and 8 pulses (−79% vs. sham) at 48 h, whereas no significant changes were detected in response to 1 pulse compared to the untreated counterpart (Figure 3B).

Cell death counting displayed a large increase following PEF stimulation over time, particularly with 20 pulses in PANC-1 and with 8 pulses in MIA PaCa-2 (Figure 3C,D). The dead-versus-total ratio provides different kinetics of cytotoxicity between PANC-1 and MIA PaCa-2 after PEFs. The mortality rate increased over time in PANC-1 cells exposed to 12 and 20 pulses (Figure 3E). Conversely, the maximum percentage of cell death was recorded at 24 h in MIA PaCa-2 cells, both in response to 4 and 8 pulses (Figure 3F). This trend was also supported by the LDH assay, which showed the highest enzymatic activity into the surrounding medium at 48 h in PANC-1 cells and comparable levels within 24 and 48 h in MIA PaCa-2 cells (Figure S2). Statistical significance is reported in Figure 3.

Taken together, these findings suggest that PEFs impair PDAC proliferation by reducing living cells and inducing cytotoxicity, with distinct temporal patterns between PANC-1 and MIA PaCa-2 cells.

### 3.4. PEFs Affect Cell Cycle Progression in PDAC Models

To provide a comprehensive overview of the investigated phenomenon, DNA content at different stages of the cell cycle was estimated by flow cytometry, and selected key cell cycle proteins were analyzed by Western blotting. PANC-1 and MIA PaCa-2 cells were stimulated with the same pulsing conditions employed for the cell growth experiments.

**Figure 4:**
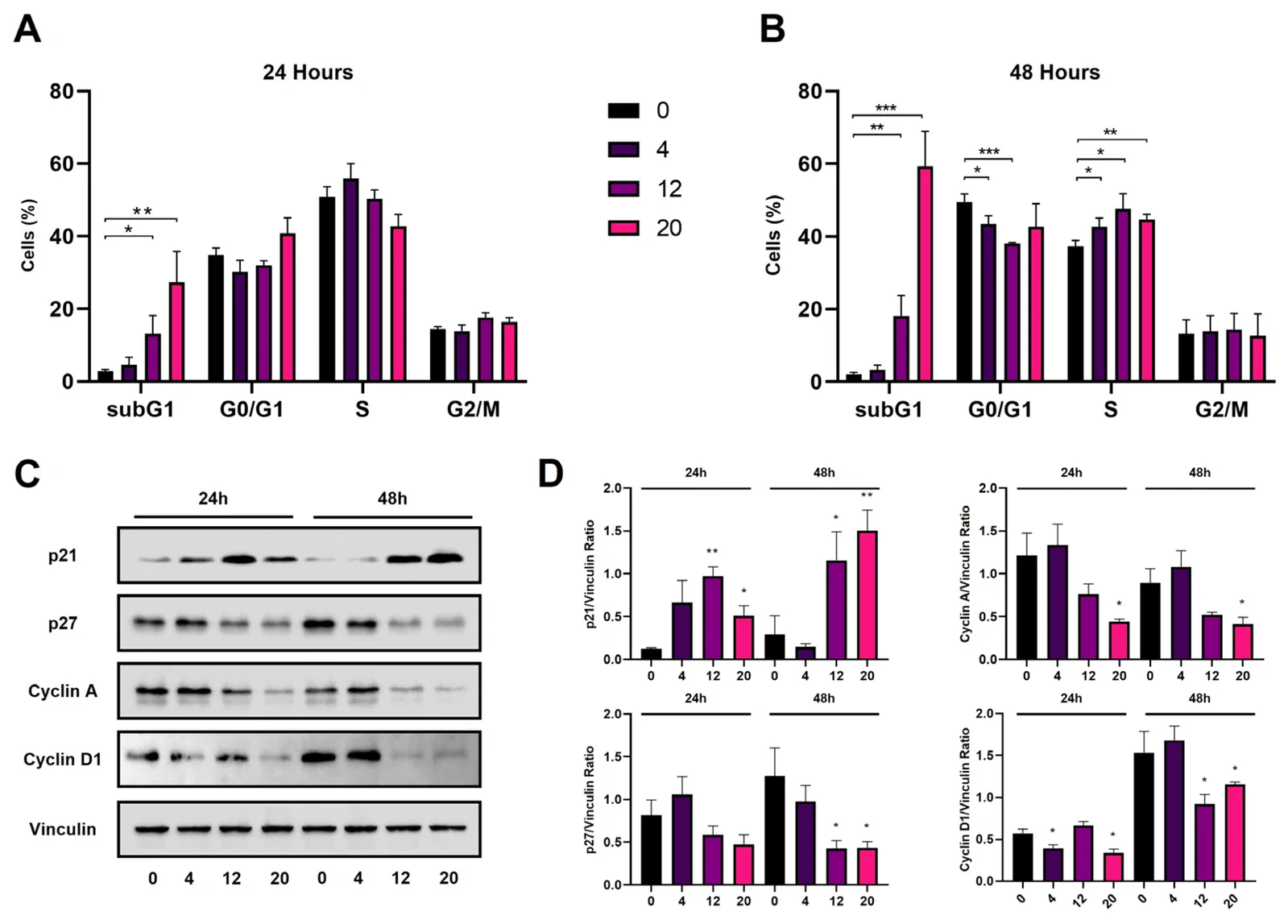
Estimation of the pulse-mediated effects on cell cycle progression in PANC-1 cells. After exposure to 4, 12, and 20 pulses (1 kV/cm), PANC-1 cells were cultured for up to 48 h. The percentage of cells in each stage of cell cycle was assessed by flow cytometry at 24 (**A**) and 48 (**B**) h after stimulation. Representative Western blot images showing the expression of p21, p27, Cyclin A and cyclin D1 are reported in (**C**). Quantitative analysis from at least three independent experiments employing vinculin as internal loading control is shown in (**D**). Horizontal axes indicate the number of applied pulses. * *p* < 0.05, ** *p* < 0.01, *** *p* < 0.001 by Welch’s *t*-test vs. sham (0 pulses).

**Figure 5:**
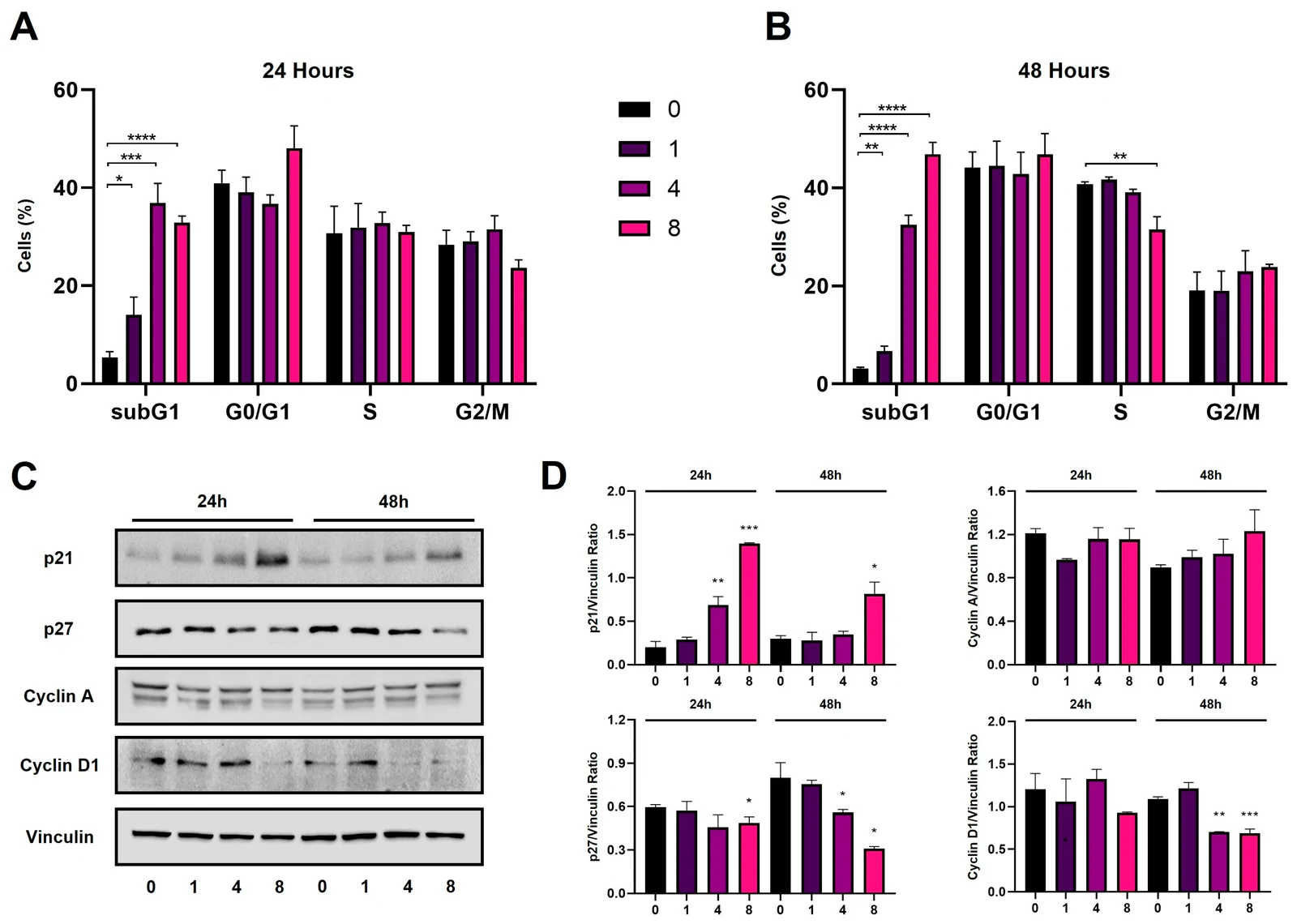
Estimation of the pulse-mediated effects on cell cycle progression in MIA PaCa-2 cells. After exposure to 1, 4, and 8 pulses (1 kV/cm), MIA PaCa-2 cells were cultured for up to 48 h. The percentage of cells in each stage of the cell cycle was assessed by flow cytometry at 24 (**A**) and 48 (**B**) h after stimulation. Representative Western blot images showing the expression of p21, p27, Cyclin A and cyclin D1 are reported in (**C**). Quantitative analysis from at least three independent experiments employing vinculin as internal loading control is shown in (**D**). Horizontal axes indicate the number of applied pulses. * *p* < 0.05, ** *p* < 0.01, *** *p* < 0.001, **** *p* < 0.0001 by Welch’s *t*-test vs. sham (0 pulses).

No significant changes compared with the sham were detected in cell cycle progression at 24 h in PANC-1 (Figure 4A), while an accumulation of cells in S-phase was observed at 48 h, irrespective of the number of pulses applied (Figure 4B). MIA PaCa-2 cells also exhibited no variations across the different stages of cell cycle at 24 h (Figure 5A), whereas at 48 h, a lower percentage of cells in S-phase was detected when 8 pulses were delivered (Figure 5B). DNA fragmentation, as indicated by subG1 peak, was detected in PDAC cells exposed to PEFs, especially in response to 12 and 20 pulses in PANC-1 cells (Figure 4A,B) and with 4 and 8 pulses in MIA PaCa-2 (Figure 5A,B). Representative histograms for all the conditions tested are reported in Figures S3 and S4 for PANC-1 and MIA PaCa-2 cells, respectively.

Furthermore, the levels of the selected proteins involved in cell cycle regulation (p21, p27, Cyclin A and Cyclin D1) were measured. An accumulation of p21 was recorded in PANC-1 cells after exposure to 12 and 20 pulses, while a decrease in Cyclin A, Cyclin D1 and p27 was observed particularly with 20 pulses, at 24 and 48 h (Figure 4C,D). Except for Cyclin A, similar changes were also detected in MIA PaCa-2 cells, where an increase in p21 was recorded in response to 8 pulses at 24 and 48 h, while p27 and Cyclin D1 appeared to decrease at 48 h following exposure to 4 and 8 pulses (Figure 5C,D). No statistically significant variations were found for most of the other conditions tested compared with the sham control.

Overall, these results point to the sub-G1 peak as a common PEF-mediated target in PDAC cells. Changes in cell cycle progression may be ascribed to specific cellular responses rather than to a specific pulsing condition.

### 3.5. PEFs Induces Apoptosis and Autophagy in PDAC Models

Since PDAC cells undergo extensive cell death following exposure to a high number of pulses, we assessed molecular markers involved in cellular stress response mechanisms, focusing mainly on apoptosis and autophagy.

**Figure 6:**
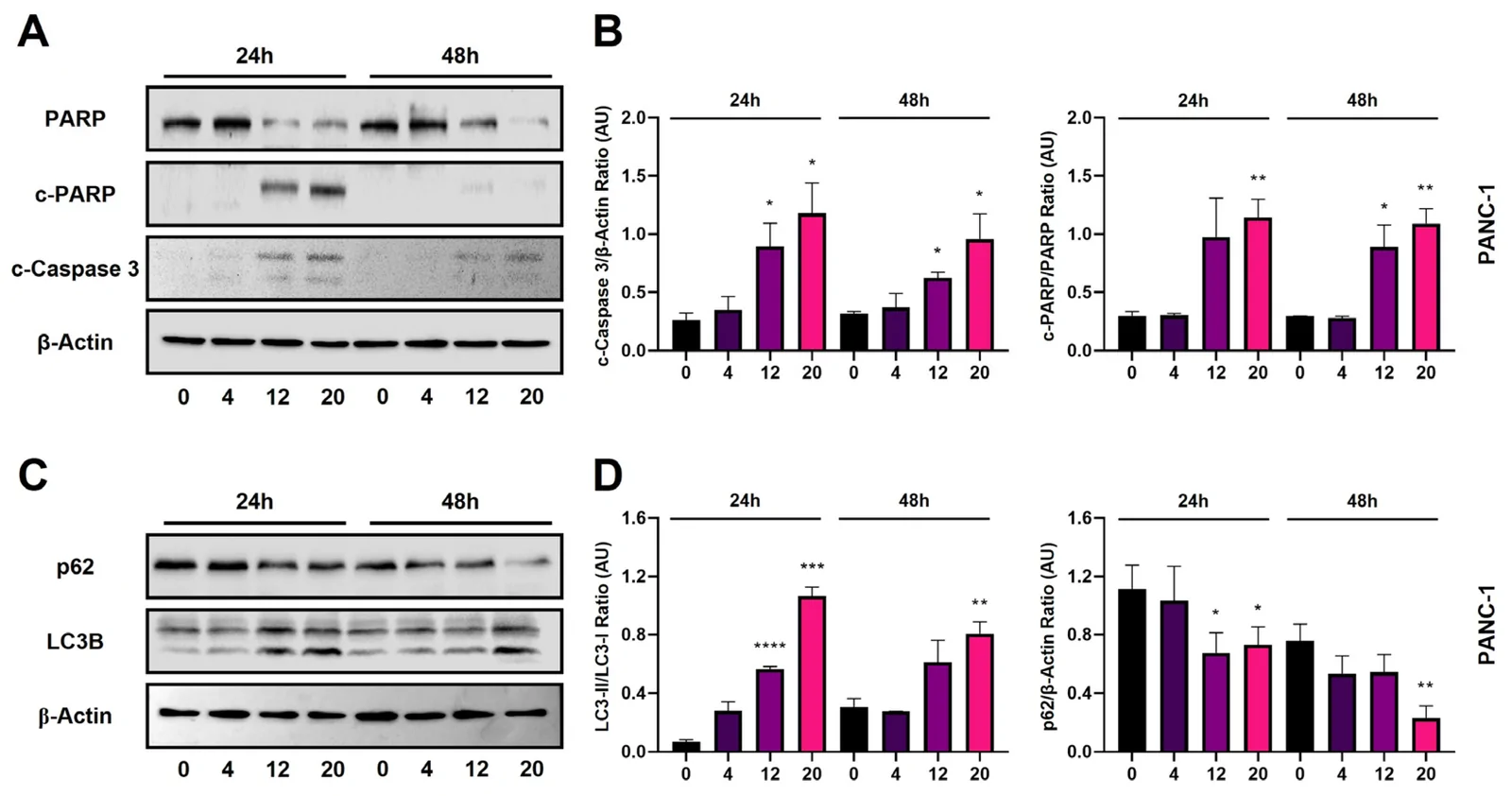
Assessment of the pulse-mediated effects on apoptosis and autophagy markers in PANC-1 cells. Following electrical stimulation with 0, 4, 12 and 20 pulses (1 kV/cm), PANC-1 cells were replaced in culture and assessed for the apoptosis and autophagy markers every 24 h. PARP, cleaved PARP (c-PARP), cleaved caspase 3 (c-Caspase 3), p62 and LC3B expressions were assessed by Western blotting. Representative Western blot images are shown in (**A**,**C**), while quantitative analyses from at least three independent experiments using β-actin as internal loading control are reported in (**B**,**D**). Horizontal axes indicate the number of applied pulses. * *p* < 0.05, ** *p* < 0.01, *** *p* < 0.001, **** *p* < 0.0001 by Welch’s *t*-test vs. sham (0 pulses).

**Figure 7:**
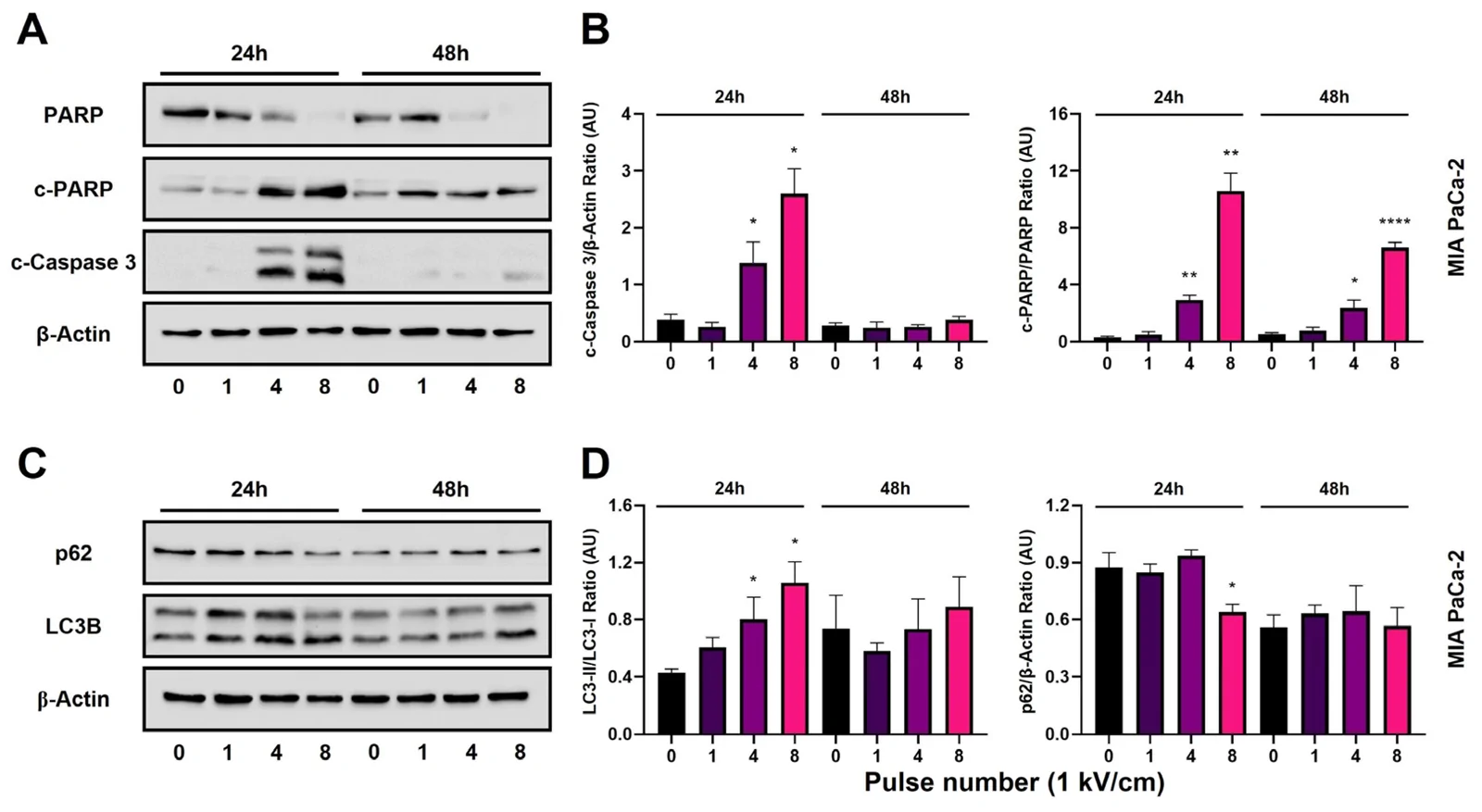
Assessment of the pulse-mediated effects on apoptosis and autophagy markers in MIA PaCa-2 cells. Following electrical stimulation with 0, 1, 4 and 8 pulses (1 kV/cm), MIA PaCa-2 cells were replaced in culture and assessed for the apoptosis and autophagy markers every 24 h. PARP, cleaved PARP (c-PARP), cleaved caspase 3 (c-Caspase 3), p62 and LC3B expression were assessed by Western blot. Representative Western blot images are shown in (**A**,**C**), while quantitative analyses from at least three independent experiments using β-actin as internal loading control are reported in (**B**,**D**). Horizontal axes indicate the number of applied pulses. * *p* < 0.05, ** *p* < 0.01, **** *p* < 0.0001 by Welch’s *t*-test vs. sham (0 pulses).

The simultaneous cleavage of caspase-3 and PARP revealed a distinct apoptosis engagement because of higher pulsing stimulation in PDAC cells. However, the timing of apoptosis induction differed between PANC-1 and MIA PaCa-2 cells, with the latter showing a statistically significant increase in cleaved caspase-3 levels at 24 h but not at 48 h, as occurred in PANC-1 (Figure 6A,B and Figure 7A,B).

The analysis of autophagy unveiled increased autophagosome formation as indicated by an elevated LC3-II/LC3-I ratio, accompanied by enhanced autophagic flux evidenced by the decreased p62 levels in both PDAC models following PEFs (Figure 6C,D and Figure 7C,D). Nevertheless, as observed for the apoptotic markers, the kinetics of autophagy induction differed slightly between PANC-1 and MIA PaCa-2 cells, as well as among PEF conditions. Specifically, 12 pulses triggered autophagy at 24 h in PANC-1, whereas 20 pulses induced autophagy at both 24 and 48 h (Figure 6C,D). In contrast, in MIA PaCa-2 cells, the induction of this catabolic process was detected only following exposure to 8 pulses at 24 h (Figure 7C,D).

Overall, these findings indicate the activation of both apoptotic and autophagic pathways in PDAC following PEF exposure, supporting their involvement in PEF-mediated cytotoxicity.

## 4. Discussion

PDAC is one of the most lethal and aggressive tumor types largely due to its resistance to conventional chemotherapeutic agents including gemcitabine, which remains the current standard of care despite its limited clinical efficacy [13,14]. In this context, EP has gained attention as a complementary strategy, and most studies have focused on its use in combination with chemotherapeutic agents, nanoparticles, or immunotherapies to enhance intracellular uptake and local tumor control [15].

In EP-based treatments, REP aims to induce transient membrane permeabilization while preserving cell viability, whereas the goal of IRE is to maximize cell death without causing thermal damage [2,16]. The transition from reversible membrane permeabilization to irreversible cell damage is increasingly recognized as a continuum rather than a sharply defined threshold. The biological outcome depends not only on the applied electrical parameters but also on the intrinsic characteristics of the target cells and the time elapsed after treatment. Therefore, defining how pulse parameters translate into distinct biological outcomes remains a major limitation for protocol optimization in both clinical and preclinical settings.

Most previous studies investigating EP in PDAC have focused on enhancing the efficacy of IRE or ECT through combination strategies, including chemotherapeutics, nanoparticles, hyperthermia, and oncolytic viruses [6,8,9,17,18,19,20,21,22,23,24,25,26]. These approaches consistently demonstrate improved cytotoxicity and tumor control, but they provide only limited information on the intrinsic biological response of PDAC cells to PEF exposure alone.

From a mechanistic perspective, EP-induced cell death has been associated with both regulated and accidental cell death pathways depending on stimulation intensity and duration [3]. However, the temporal dynamics linking membrane permeabilization, metabolic collapse, and the activation of downstream stress pathways remain poorly characterized, particularly in human PDAC models.

In this study, we specifically addressed the intrinsic response of two widely used PDAC cell lines to microsecond PEFs applied in the absence of adjunct therapies, allowing the effects of the electric stimulus itself to be evaluated. The results show that, within 5 min after high-intensity PEFs, rapid loss of membrane integrity and cell death occurred at cell line-dependent exposure conditions (16 pulses at 1 kV/cm and 30 pulses at 1 kV/cm for PANC-1 and MIA PaCa-2, respectively, Figure 1 and Figure S1). The rapid onset of cell death following high-intensity PEF exposure supports the hypothesis that membrane damage exceeded the cellular repair capacity, leading to the immediate loss of homeostasis, which is consistent with accidental cell death mechanisms [3]. PANC-1 cells displayed lower tolerance to membrane disruption compared with MIA PaCa-2 cells, suggesting differential susceptibility to EP-induced homeostatic collapse. While previous studies have reported variability in EP sensitivity across tumor types and experimental setups [3,27,28], our findings specifically highlight intrinsic heterogeneity within PDAC cells under the same pulsing conditions despite their common histological origin. Moreover, the analysis of flow-cytometry data highlighted that heterogeneous cell responses coexist within the same population, with some cells remaining viable, others permeabilized, and others irreversibly damaged (Figure 1 and Figure S1). Importantly, rather than a sharp threshold between REP and IRE, our data support a graded transition between permeabilized and irreversibly damaged states and further demonstrates that this transition occurs at different exposure conditions in PANC-1 and MIA PaCa-2 cells.

The delayed outcomes, observed at 24 h and 48 h after PEF exposure, further emphasize differences between PANC-1 and MIA PaCa-2, especially in terms of cell viability, where a pulse number-dependent response was observed in both PDAC cells, with greater sensitivity in MIA-PaCa-2 (Figure 2).

The analysis of cell cycle distribution revealed S-phase accumulation in PANC-1 at 48 h (Figure 4 and Figure S3). In a study investigating the role of DNA damage repair inhibitors, Long and colleagues recently proved that REP induces an arrest at the S-phase in PANC-1 cells, which is consistent with our findings [29]. On the contrary, the percentage of cells in S-phase was lower 48 h after exposure to 8 pulses than in untreated MIA PaCa-2 cells (Figure 5 and Figure S4). These contrasting results highlight the relevance of experimental variables in determining the impact of PEFs on cell cycle progression. Due to their dissimilar sensitivity, PANC-1 and MIA PaCa-2 were exposed to different pulse numbers before being analyzed for cell cycle distribution. The chance that PEFs settings may have disparate outcomes on cell cycle progression is supported by robust scientific evidence, both in PDAC and in other cancers. Imran and colleagues observed that IRE induces G0/G1 arrest at field strengths ranging from 0.2 to 0.6 kV/cm in murine pancreatic adenocarcinoma cell lines [27]. Interestingly, treating the same cells with higher electric fields ranging between 1.0–2.0 kV/cm provoked an increase in the S-phase. Conflicting results were also obtained in HeLa cells, where exposure to 1.0 kV/cm caused G1/S blockage, while treatments with field strengths of 2.0–2.5 kV/cm increased G2 phase accumulation [28,30]. Another relevant aspect that should not be underestimated is the intrinsic features of each cell line. Although PANC-1 and MIA PaCa-2 cells share genetic abnormalities, they are morphologically and biologically different, as also supported by their sensitivity to PEFs. MIA PaCa-2 cells are more mesenchymal-like, often showing a higher proliferation rate and targeted therapy sensitivity, whereas PANC-1 cells are more epithelial-like, displaying greater stemness potential [31]. The expression pattern of cell cycle-related proteins appears very close between PANC-1 and MIA PaCa-2 after PEFs stimulation. Except for Cyclin A, we observed an increase in p21 together with a concomitant decrease in both p27 and Cyclin D1 because of high pulsing conditions in PDAC cells. Besides regulating cell cycle progression, these proteins are involved in many other cellular functions, including apoptosis, DNA repair and epithelial–mesenchymal transition [32,33,34]. Therefore, discrepancies in cell cycle progression and similarities in cell cycle proteins may coexist precisely because of the multifaceted role played by the latter.

The appearance of subG1 peak, along with both TBEM and LDH results, appears consistent with a direct cytotoxic action that occurs when PDAC cells are exposed to PEFs (Figure 3 and Figure S2). Mechanistically, the simultaneous cleavage of both caspase-3 and PARP provides evidence of the involvement of apoptosis as one of the death mechanisms induced by PEFs in PDAC cells (Figure 6 and Figure 7). Together with the necrosis, apoptosis represents the main cause of cell death following PEF-mediated tumor ablation [35]. In mouse models of prostate cancer, Kim and collaborators highlighted the appearance of cleaved caspase-3 bands in the IRE-treated groups with an increasingly trend as the applied voltage increased [36]. IRE also induced caspase-3 cleavage in murine models of PDAC [37]. Remarkably, combining IRE with chemotherapy drugs also resulted in a significant increase in the mRNA levels of caspase-3. Although our results are consistent with these findings, there is no shortage of conflicting reports about apoptosis’ involvement in PEFs responses in pancreatic tumors specifically [35]. José et al. detected no caspase-3-positive cells after IRE stimulation in PDAC in vivo models [38]. Both technical and experimental issues could easily rationalize these inconsistencies. From a technical perspective, it is widely known that changing the type of electrodes used, as well as the number and repetition rate of applied pulses, can result in different outcomes, especially in highly heterogenous diseases like cancer. Moreover, IRE protocols may affect the kinetics of cellular response, and thus the timing of analysis becomes essential to portray the dynamics of apoptosis.

Following high-pulse-number stimulation, cytotoxic effects were also associated with an increased LC3-II/LC3-I ratio and decreased p62 levels in both PANC-1 and MIA PaCa-2 cells (Figure 6 and Figure 7). Considering LC3-II a good indicator of autophagosome formation and p62 as a marker of degradation activity [39], PEFs may lead to the induction of autophagy in PDAC cells. Autophagy is a key catabolic process that helps cells to preserve homeostasis against both intracellular and environmental stress. The potential involvement of autophagy in the effects mediated by PEFs is still relatively unexplored in cancer. In agreement with our results, Ye and colleagues demonstrated that IRE induces LC3 expression followed by p62 decrease in murine pancreatic cancer cell line xenograft mouse models [40]. They also identified autophagy as a survival mechanism against IRE, as both pharmacological and genetic inhibition enhanced the sensitivity of pancreatic cancer cells to sublethal PEFs. However, the impact of PEFs on autophagy may differ depending on the degree of EP. Condello and coworkers observed that REP did not change p62 and LC3 levels in human hypopharyngeal and tongue carcinoma cell lines, while ECT with Mitomycin C enhanced apoptosis by inhibiting autophagy [41]. Interestingly, autophagic activity is usually upregulated in PDAC cells, where it promotes tumor progression and metastasis [42]. When unable to restore homeostasis, autophagy can activate different death programs, including apoptosis [43]. Autophagy and apoptosis share common signaling pathways and regulatory proteins that allow a mutual control at cellular levels [39]. Regrettably, protein analyses were performed on bulk cell populations, which included both viable and non-viable cells, preventing the definitive attribution of autophagy to specific subpopulations. Future studies using cell sorting approaches or the pharmacological modulation of autophagy and apoptosis will be required to clarify their functional roles in EP-induced cell fate decisions.

Special efforts should also be focused on the assessment of molecular mechanisms underlying the EP-mediated effects in PDAC cells. Defining the impact of EP on the principal signaling pathways and molecular mechanisms implicated in the initiation and progression of PDAC might represent an initial approach to better characterize the biological effect of the EP. Special emphasis should be placed on hallmarks of PDAC, including PI3K/Akt, RAF/MEK/ERK, TGF-β, Hedgehog (Hh), Notch and Wnt/β-Catenin pathways [44]. Remarkably, most of this intracellular signaling is also actively involved in the crosstalk between autophagy and apoptosis [39].

Although these findings demonstrate the differential susceptibility of PDAC cell lines under controlled in vitro conditions, clinical translation requires further validation. In vivo tissues exhibit heterogeneous conductivity, extracellular matrix composition, vascular perfusion, immune interactions, and complex electric field distributions that cannot be fully recapitulated in monolayer cultures. Therefore, additional validation in three-dimensional models and animal studies will be required before extrapolating these findings to clinical applications.

## 5. Conclusions

This study investigated the response of PDAC cells to microsecond pulsed electric fields (PEFs) in the absence of adjunctive treatments. The results demonstrate that PEFs alone induce a spectrum of biological effects, ranging from immediate membrane permeabilization and death to delayed alterations in viability, growth and stress responses. These effects were strongly dependent on pulse delivery conditions and differed between the two PDAC cell models, emphasizing the reliance of biological responses on both PEF parameters and target cells. Therefore, the future optimization of EP-based therapeutic protocols should consider PEF parameters alongside the tumor recipient features.

## Figures and Tables

**Table 1:** Pulsing conditions adopted for each biological assay in PANC-1 and MIA PaCa-2 cell lines. CAM/PI, Calcein-AM/Propidium Iodide; MTT, 3-(4,5-dimethylthiazol-2-yl)-2,5-diphenyltetrazolium bromide; TBEM, Trypan Blue Exclusion Method; LDH, Lactate Dehydrogenase; WB, Western Blotting.

Biological Assay	Line	Voltage/Distance [kV/cm]	Pulse Number
CAM/PI	PANC-1	1	4, 8, 16, 20, 30
		1.5, 2, 2.5	30
		2.5	50
	MIA PaCa2	1	1, 4, 8, 12, 16, 20, 30
		1.5, 2, 2.5	30
		2.5	50
MTT	PANC-1	1	1, 4, 8, 12, 16, 20, 30
	MIA PaCa2	1	1, 4, 8, 12, 16, 20, 30
TBEM, LDH Release, PI staining, WB	PANC-1	1	4, 12, 20
	MIA PaCa2	1	1, 4, 8

## Data Availability

The raw data supporting the conclusions of this article will be made available by the authors on request.

## Supplementary Materials

The following supporting information can be downloaded at https://www.mdpi.com/article/10.3390/bioengineering13091056/s1.

## Abbreviations

The following abbreviations are used in this manuscript:

CAM	Calcein acetoxymethyl ester
ECT	Electrochemotherapy
EP	Electroporation
IRE	Irreversible electroporation
LDH	Lactate dehydrogenase
MTT	(3-(4,5-dimethylthiazol-2-yl)-2,5 diphenyltetrazolium bromide)
PARP	Poly (ADP-ribose) polymerase
PDAC	Pancreatic ductal adenocarcinoma
PEF	Pulsed electric field
PI	Propidium iodide
REP	Reversible electroporation
RT	Room temperature
TBEM	Trypan blue exclusion method
