# Supplementary material for: Parameter-Dependent Electroporation and Cellular Responses Induced by Microsecond Pulsed Electric Fields: Different Sensitivity of Two Pancreatic Ductal Adenocarcinoma Cell Lines
Source: Bioengineering (Basel). 2026 Sep 11;13(9):1056. doi: 10.3390/bioengineering13091056 (PMC13603243; doi:10.3390/bioengineering13091056)
Supplement: Supplementary file 1 [file bioengineering-13-01056-s001.zip › bioengineering-4513188-supplementary.pdf]

Supplementary figures

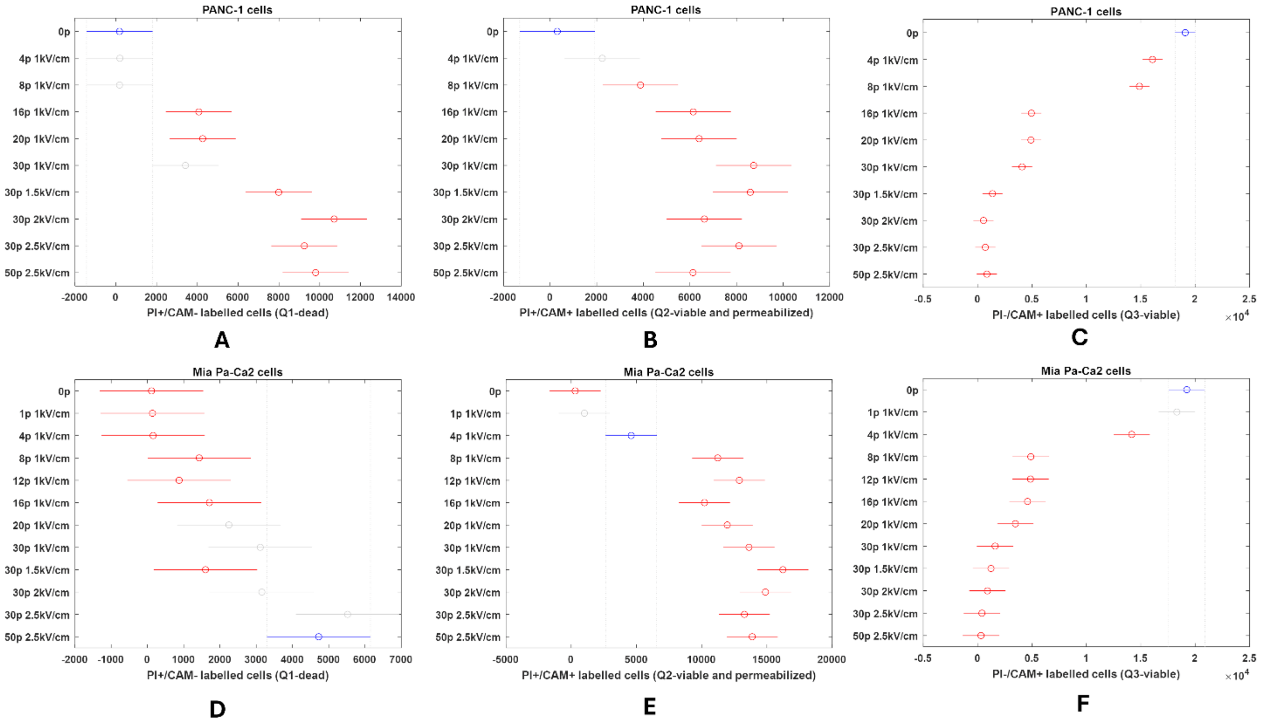

Figure S1. One-way ANOVA test followed by Tuckey post-hoc test for CAM/PI analysis in PANC-1 (A-C) and in MIA PaCa-2 (D-F) cells.

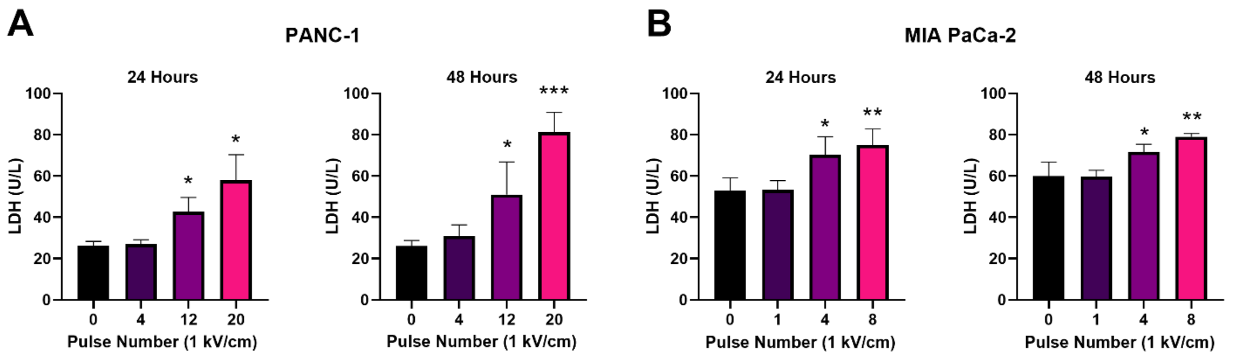

Figure S2. LDH enzymatic activity in exhausted PANC-1 (A) and MIA PaCa-2 (B) media. * $p < 0.05$, ** $p < 0.01$, *** $p < 0.001$ by Welch's t-test.

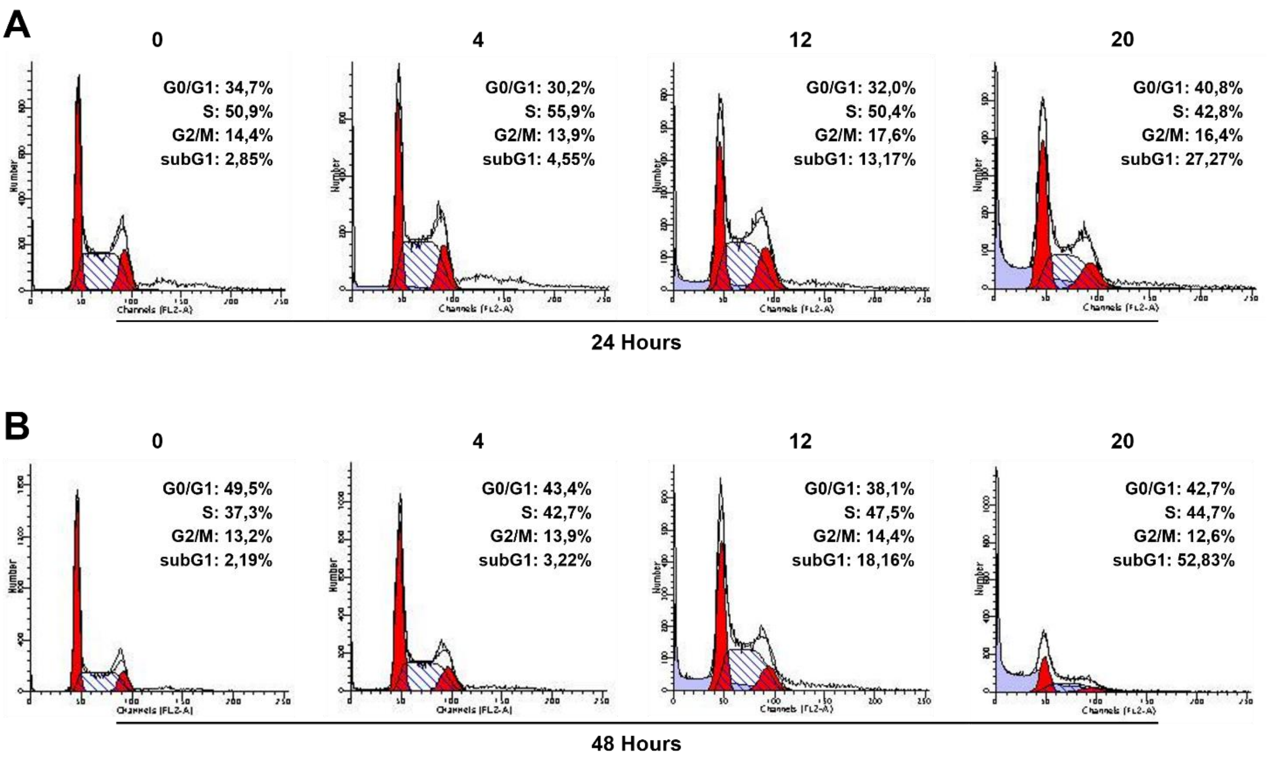

Figure S3. Representative cell cycle profile achieved in PANC1 cells exposed and not (0) at 4, 12, and 20 pulses (1 kV/cm) for 24 (A) and 48 (B) hours.

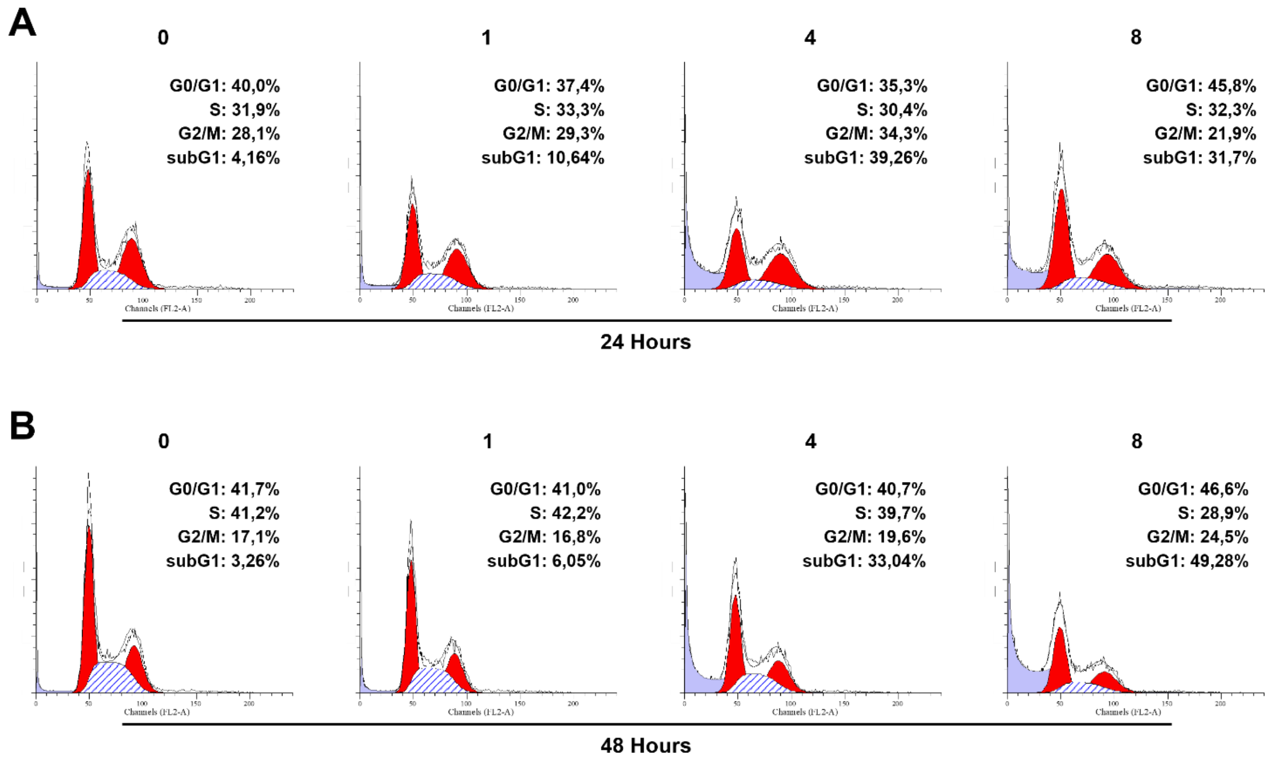

Figure S4. Representative cell cycle profile achieved in MIA PaCa-2 cells exposed and not (0) at 1, 4, and 8 pulses (1 kV/cm) for 24 (A) and 48 (B) hours.
